# Differences in soil physicochemical properties and rhizosphere microbial communities of flue-cured tobacco at different transplantation stages and locations

**DOI:** 10.3389/fmicb.2023.1141720

**Published:** 2023-04-20

**Authors:** Leifeng Zhao, Yuansheng He, Yuanxian Zheng, Yinlian Xu, Shoujie Shi, Meixun Fan, Shaolong Gu, Guohong Li, Wajie Tianli, Jiming Wang, Junying Li, Xiaopeng Deng, Xiaolin Liao, Jun Du, Fuzhao Nian

**Affiliations:** ^1^College of Tobacco Science, Yunnan Agricultural University, Kunming, Yunnan, China; ^2^Lincang Branch Company of Yunnan Tobacco Company, Lincang, Yunnan, China; ^3^Yunnan Academy of Tobacco Agricultural Sciences, Kunming, Yunnan, China; ^4^College of Food Science and Technology, Yunnan Agricultural University, Kunming, Yunnan, China; ^5^Institute of Plant Nutrition, Agricultural Resources and Environmental Science, Henan Academy of Agricultural Sciences, Zhengzhou, China

**Keywords:** flue-cured tobacco, soil, rhizosphere microbiota, microbiome, metabolism pathway

## Abstract

Rhizosphere microbiota play an important role in regulating soil physical and chemical properties and improving crop production performance. This study analyzed the relationship between the diversity of rhizosphere microbiota and the yield and quality of flue-cured tobacco at different transplant times (D30 group, D60 group and D90 group) and in different regions [Linxiang Boshang (BS) and Linxiang ZhangDuo (ZD)] by high-throughput sequencing technology. The results showed that there were significant differences in the physicochemical properties and rhizosphere microbiota of flue-cured tobacco rhizosphere soil at different transplanting times, and that the relative abundance of Bacillus in the rhizosphere microbiota of the D60 group was significantly increased. RDA and Pearson correlation analysis showed that *Bacillus*, *Streptomyces* and *Sphingomonas* were significantly correlated with soil physical and chemical properties. PIGRUSt2 function prediction results showed that compared with the D30 group, the D60 group had significantly increased metabolic pathways such as the superpathway of pyrimidine deoxyribonucleoside salvage, allantoin degradation to glyoxylate III and pyrimidine deoxyribonucleotides *de novo* biosynthesis III metabolic pathways. The D90 group had significantly increased metabolic pathways such as ubiquitol-8 biosynthesis (prokaryotic), ubiquitol-7 biosynthesis (prokaryotic) and ubiquitol-10 biosynthesis (prokaryotic) compared with the D60 group. In addition, the yield and quality of flue-cured tobacco in the BS region were significantly higher than those in the ZD region, and the relative abundance of Firmicutes and Bacillus in the rhizosphere microbiota of flue-cured tobacco in the BS region at the D60 transplant stage was significantly higher than that in the ZD region. In addition, the results of the hierarchical sample metabolic pathway abundance map showed that the PWY-6572 metabolic pathway was mainly realized by *Paenibacillus*, and that the relative abundance of flue-cured tobacco rhizosphere microbiota (*Paenibacillus*) participating in PWY-6572 in the D60 transplant period in the BS region was significantly higher than that in the ZD region. In conclusion, different transplanting periods of flue-cured tobacco have important effects on soil physical and chemical properties and rhizosphere microbial communities. There were significant differences in the rhizosphere microbiota and function of flue-cured tobacco in different regions, which may affect the performance and quality of this type of tobacco.

## 1. Introduction

Good soil conditions are needed for high-quality tobacco production (Mallikharjuna Rao et al., 2016; Begum et al., 2021; Shen et al., 2022). To achieve sustainable tobacco production, soil quality must be improved (Wang et al., 2019; Yan et al., 2019; Wang et al., 2022). However, due to the extensive use of chemical fertilizer and a series of bad farming measures in tobacco planting regions, the soil microbial abundance and diversity have been reduced, leading to soil acidification, hardening, fertility decline and other outstanding problems, which not only seriously affect crop yield but also adversely affect the sustainable development within the ecosystem and the green environment (Wang Q. et al., 2017; Kavamura et al., 2018; Li et al., 2019). The root system represents the most direct “contact” plants have with the soil (Rich and Watt, 2013). Microbiota can affect the growth, development and morphological characteristics of flue-cured tobacco root systems by improving the physical and chemical properties of soil. Rhizosphere microbiota are an important part of the soil ecosystem and play an important role in the formation and development of soil, the soil nutrient cycle, fertility evolution and plant growth (Niu et al., 2016, 2017; Zheng et al., 2021). The term rhizosphere microbiota refers to the microbiota closely attached to the rhizosphere soil particles, including bacteria and fungi, with bacteria accounting for more than 90% of the total number of microbiota. As a complex ecosystem, the rhizosphere microbiota play a key role in regulating terrestrial carbon dynamics, nutrient cycling and plant productivity (Qu et al., 2020; Zheng et al., 2021; Alegria Terrazas et al., 2022; Jacquiod et al., 2022).

The interaction modes of rhizosphere microbiota include nutritional interdependencies, enhanced dispersion and quorum sensing. Nutritional dependency is an interactive relationship of nutrient-deficient microbiota in the community that compensate for their own metabolic defects by exchanging extracellular metabolites and promoting their own growth (Zengler and Zaramela, 2018; Du et al., 2022; Iven et al., 2022). The *Bacillus cereus* metabolite peptidoglycan can promote the growth of rhizosphere microbiota. This form of interaction can expand the basic niches of microbiota, enabling them to survive in a nutrient-poor environment (Peterson et al., 2006; Hassani et al., 2018). This interaction strategy plays an important role in building soil microbial networks. Quorum sensing is a signal transduction mechanism. It depends on the population density of the microbiota and regulates the expression, metabolism and other physiological processes of specific genes of bacterial members. In addition to quorum sensing mechanisms, many microbial compounds, such as volatile organic compounds, oxalic acid, glucose, etc., can also act as signal molecules to trigger microbial interactions (Schaefer et al., 2013; Mukherjee and Bassler, 2019). These interactions involve many microbiota, and the sum of the interactions determines the composition and function of the rhizosphere microbiome.

In addition, rhizosphere microbiota and plants are symbiotic, interacting and promoting each other through the plant rhizosphere. During plant growth, inorganic and organic substances secreted from the rhizosphere provide nutrients for microbiota or change soil environmental conditions, thus regulating the growth and structural composition of rhizosphere microbiota (Jin et al., 2019; Kong et al., 2020; Feng et al., 2021). Microbes gather around the rhizosphere, release carbon dioxide or metabolize acid by respiration, which helps to dissolve insoluble minerals and increase the absorption of phosphorus and other mineral elements by plants. Microbes synthesize a variety of proteases to degrade and transform organic matter and provide effective nutrients for plants (Huang et al., 2019). At the same time, microbiota can also produce amino acids, vitamins, antibiotics, and growth stimulators, which inhibit the propagation of pathogenic bacteria and promote plant growth (Renu Gupta et al., 2019). As an important indicator of soil ecosystems, rhizosphere microbiota are closely related to crop yield and quality. Increasing evidence indicates that the plant rhizosphere microbiome plays an important role in promoting host growth, health and tolerance. Excavating and utilizing the beneficial members of the rhizosphere microbiome and processing them into microbial inoculants or fertilizers is a very promising new pesticide and fertilizer preparation avenue (Hu et al., 2016). The transplanting period is the main means to regulate the growth of tobacco in suitable climatic conditions and to ensure that the whole tobacco field growth period is in suitable light, temperature, precipitation and other climatic conditions. A suitable transplanting period is one of the important links in the production of high-quality tobacco leaves (Sreeramulu et al., 2000; Gu et al., 2012). The results of previous studies on the transplanting period are quite consistent. If the transplanting is too early, the tobacco plant is prone to early flowering when it encounters low temperature, and the yield and quality of the tobacco leaves will be reduced. Tobacco plants cannot complete their normal growth cycle when they are transplanted too late and meet high temperatures (Sinha et al., 1984). There are many microorganisms and nutrients in the soil, which is one of the important ecological environmental conditions that affect the quality of tobacco leaves. Under the appropriate ecological conditions, selecting a good soil structure and nutrient status is the key factor in improving the quality of tobacco leaves (Richardson and Simpson, 2011; Hemkemeyer et al., 2021). However, there are few studies on the effects of different transplanting periods on the microbial community and physical and chemical properties of flue-cured tobacco rhizosphere soil. In this study, 16S rRNA gene sequencing technology was used to study the effects of different transplant times and different regions on the yield and quality of flue-cured tobacco, the physical and chemical properties of rhizosphere soil, and the microbial community structure of rhizosphere soil to provide a basis for improving the quality and performance and promoting the sustainable development of flue-cured tobacco in Southwest China.

## 2. Materials and methods

### 2.1. Experimental regions and soil physicochemical properties

Since 2020, this experiment has been carried out in Linxiang Boshang and Linxiang Zhangduo, Lincang City, Yunnan Province. The altitude, longitude and latitude of the test plot and soil agrochemical properties are shown in Supplementary Table S1. The flue-cured tobacco variety was Yunyan 87.

### 2.2. Experimental design

Two fields in the Linxiang Boshang (BS) and Linxiang Zhangduo (ZD) experimental regions were selected. In winter and spring, the fields were not replanted with rape or maize in winter and spring, and the land was prepared after the rape and maize were harvested. The base fertilizer was 600 kg/hm^2^ (N-P_2_O_5_-K_2_O = 12–14-24) before transplanting and 360 kg/hm^2^ (N-P_2_O_5_-K_2_O = 15–0-30) after transplanting. Other production measures were the same as in local conventional management, but no herbicides or other plant or soil regulators were applied. Each treatment was replicated three times for a total of 36 plots, with 300 tobacco plants planted in each plot at a row spacing of 120 cm * 50 cm. It should be noted that in this study, we first analyzed the differences in soil physical and chemical properties and microbial composition in different transplant periods (D30: Day 30 transplant, D60: Day 60 transplant and D90: Day 90 transplant). Then, the differences in microbial communities, economic value and production performance in the rhizosphere soil of flue-cured tobacco in the Boshang and Zhangduo regions were compared and analyzed.

### 2.3. Sample collection

At 30, 60 and 90 days after transplanting the flue-cured tobacco plant, the quincunx five-point sampling method was used to cut and collect the soil at the root of the flue-cured tobacco plant. For sample collection, the tobacco plant was uprooted, the 2–3 cm soil layer above was removed with a circular knife, the large pieces of soil were removed by shaking the roots, and the small soil particles attached to the roots were collected and put into an aluminum box to serve as rhizosphere soil samples. The aluminum box was taken back to the laboratory to determine the physicochemical properties and the diversity of microbiota in the rhizosphere soil.

### 2.4. Physicochemical properties of the flue-cured tobacco rhizosphere soil

At 30, 60, and 90 days after transplanting, the rhizosphere soil was collected by the quincunx 5-point sampling method and then dried and sieved naturally. Determination of soil pH (potentiometric method with water/soil ratio of 2.5:1), organic matter (potassium dichromate capacity external heating method), available nitrogen (alkali hydrolysis diffusion method), available phosphorus (NaHCO_3_ extraction molybdenum antimony resistance colorimetry method), and available potassium (NH_4_OAC extraction flame photometric method) was conducted (Ferreras et al., 2006; Ma et al., 2020). After curing, the tobacco was graded and harvested according to the 42 level national flue-cured tobacco grading standard, and the yield was calculated according to the varieties. The yield, output value, average price and proportion of medium- and top-grade tobacco were calculated according to the purchase price of local flue-cured tobacco.

### 2.5. Rhizosphere microbiota of the flue-cured tobacco plants

#### 2.5.1. Extraction of genomic DNA from the flue-cured tobacco rhizosphere microbiota

The soil microbial genomic DNA (gDNA) was extracted with an E.Z.N.A. TM kit, then the extracted genomic DNA was detected with 0.8% agarose gel electrophoresis, and the DNA was quantified with a NanoDrop 2000.

#### 2.5.2. 16S rRNA PCR amplification of tobacco rhizosphere soil bacteria

TransGen AP221-02: TransStart Fastpfu DNA Polymerase was used for PCR. The primer sequences were as follows: 338F: ACTCCTACGGGGAGCA, 806R: GGACTACHVGGGTWTCTAAT. The PCR system consisted of the following reaction volume: 25 μl, 12.5 μl 2 × Taq-PCR-MasterMix, 3 μl BSA (2 ng/μL), 1 μl (5 μmol/l) of each primer, 2 μl template DNA, and 5.5 μl ddH_2_O, and was amplified on the Mastercycler Gradient PCR (Eppendorf, Germany). The circulation parameter was 95°C for 5 min, then it was cycled at 95°C for 45 s, 55°C for 50 s, 72°C for 45 s, and finally it was prolonged at 72°C for 10 min. Each sample was repeated 3 times. The AxyPrepDNA gel recovery kit was used to cut and recover the PCR products, and Tris–HCl elution was performed. The PCR products of the same sample were mixed and detected by 2% agarose gel electrophoresis.

#### 2.5.3. Preparation of sequencing library and high-throughput sequencing

The TruSeq Nano DNA LT Library Prep Kit from the Illumina Company was used, and the sequencing library was prepared according to the manufacturer’s instructions. Before sequencing, the Agilent High Sensitivity DNA Kit was used to inspect the library, and the qualified library used the Quantit PicoGreen dsDNA Assay Kit to detect and quantify the library on the Promega QuantiFluor fluorescence quantitative system. The Illumina MiSeq sequencing platform was used to perform double-ended sequencing on the V3-V4 region of the library according to the operation instructions.

#### 2.5.4. Bioinformatics analysis

First, the original high-throughput sequencing offline data were preliminarily screened according to the sequence quality. The problem samples were retested and retested. The original sequence passing the quality preliminary screening was divided into libraries and samples according to the index and barcode information, and the barcode sequence was removed. Sequence denoising and OTU clustering were carried out according to the QIIME2 DADA2 analysis process. Through statistics on the ASV/OTU table after leveling, the specific composition table of the microbial community in each sample at each classification level was obtained. With this table, we were able to first calculate the number of taxa contained in different samples at each classification level. The alpha diversity level of each sample was evaluated according to the distribution of ASV/OTUs in different samples. Alpha diversity refers to the diversity of a specific region or ecosystem. The diversity index includes the Chao1 index, observed specifications index, Simpson index and Shannon index, which reflect the richness and diversity of samples. At the ASV/OTU level, the distance matrix of each sample was calculated, the difference and significance of beta diversity among different samples (groups) was measured by unconstrained sorting combined with corresponding statistical test methods, and the PICRUSt method was used to predict the functional potential of the microbial population based on 16S rRNA sequence (Douglas et al., 2020).

### 2.6. Statistical analysis

SPSS 24.0 was used for statistical analysis of the data differences. A visual analysis of the data was performed using GraphPad Prism 8.0. Statistical analyses of two groups and three groups were performed by Student’s *t* test and one-way ANOVA, respectively. * indicates a significant difference, *p* < 0.05; ** indicates a very significant difference, *p* < 0.01; *** indicates an extremely significant difference, *p* < 0.001; NS indicates that there is no significant difference between the data, *p* > 0.05.

## 3. Results

### 3.1. Physicochemical properties of the flue-cured tobacco rhizosphere soil

**Figure 1:**
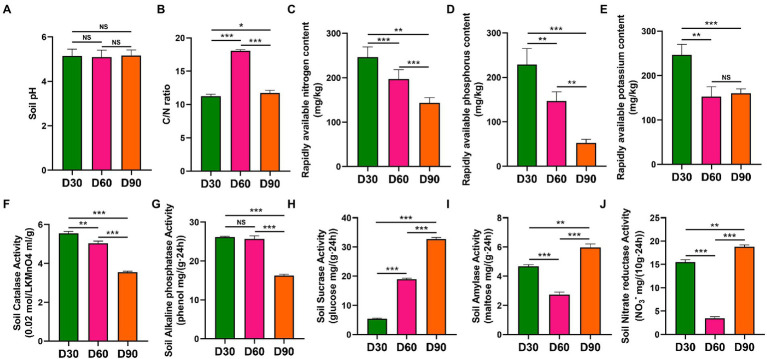
Physicochemical properties of rhizosphere soil of flue-cured tobacco at different transplantation stages. **(A)**. pH of rhizosphere soil of the flue-cured tobacco. **(B)**. C/N ratio of rhizosphere soil of the flue-cured tobacco. **(C)**. Rapidly available nitrogen content of rhizosphere soil of the flue-cured tobacco. **(D)**. Rapidly available phosphorus content of rhizosphere soil of the flue-cured tobacco. **(E)**. Rapidly available potassium content of rhizosphere soil of the flue-cured tobacco. **(F)**. Catalase activity of rhizosphere soil of flue-cured tobacco. **(G)**. Alkaline phosphatase activity of rhizosphere soil of flue-cured tobacco. **(H)**. Sucrase activity of rhizosphere soil of flue-cured tobacco. **(I)**. Amylase activity of rhizosphere soil of flue-cured tobacco. **(J)**. Nitrate reductase activity of rhizosphere soil of the flue-cured tobacco.

In the Boshang and Zhangduo flue-cured tobacco rhizosphere soil, there was no significant difference in pH values in flue-cured tobacco rhizosphere soil among the D30, D60, and D90 groups (*p* > 0.05) (Figure 1A). The C/N ratio of D60 in the rhizosphere soil was significantly higher than that in the other two time nodes (*p* < 0.001) (Figure 1B). The contents of available nitrogen, available phosphorus, available potassium, catalase and alkaline phosphatase in the rhizosphere soil decreased with the increase in transplanting time (*p* < 0.01 or *p* < 0.001) (Figures 1C–G). The activity of soil sucrase in the rhizosphere soil increased with the increase in transplanting time (*p* < 0.001) (Figure 1H). The amylase activity and nitrate reductase activity in the rhizosphere soil decreased first and then increased with the increase in transplanting time (*p* < 0.001) (Figures 1I,J).

### 3.2. Alpha and beta diversity analysis of the flue-cured tobacco rhizosphere microbiota at different transplantation stages

**Figure 2:**
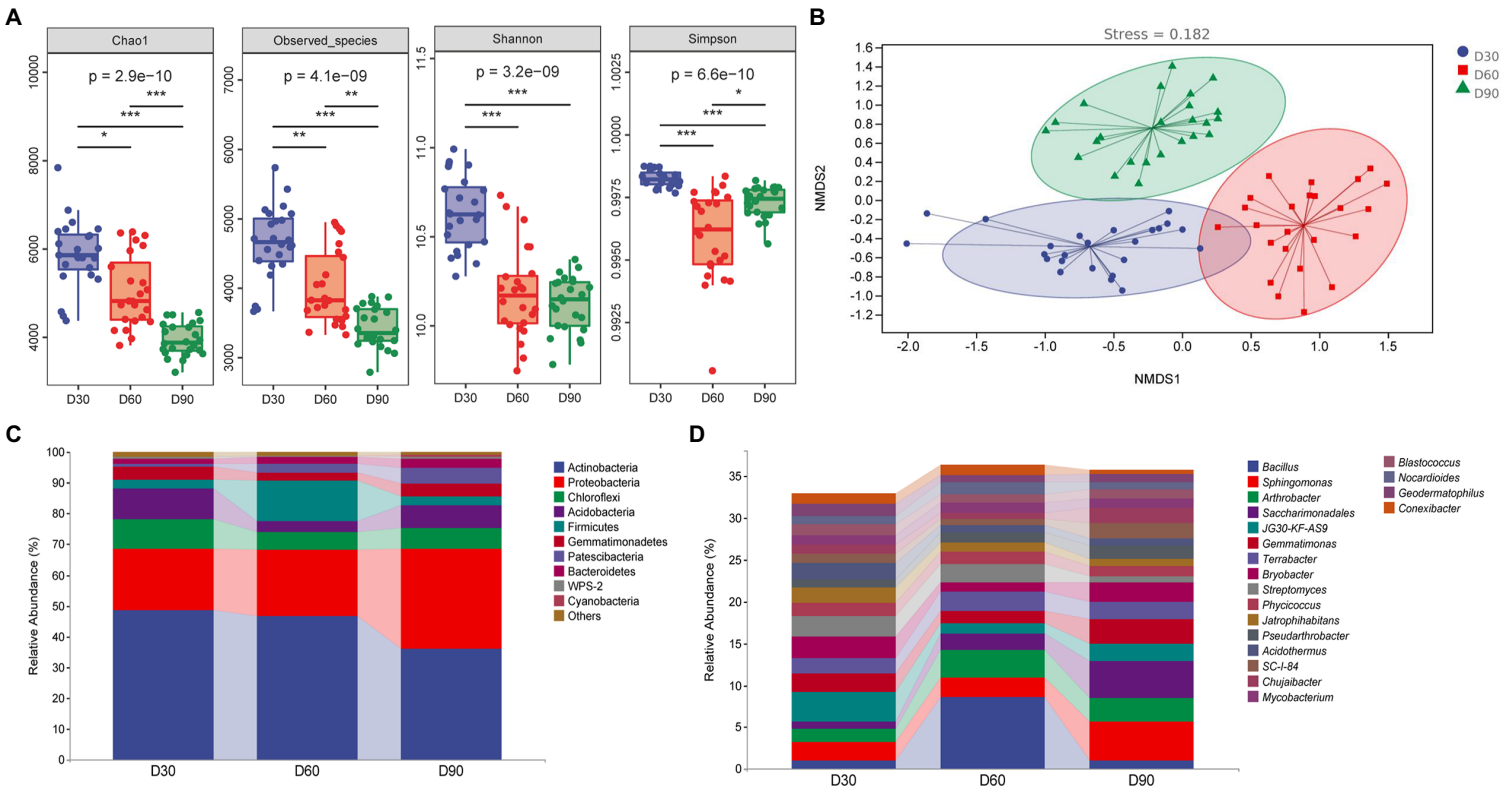
Diversity analysis of the rhizosphere microbiota of flue-cured tobacco at different transplantation stages. **(A)**. Alpha diversity of the rhizosphere microbiota of flue-cured tobacco. **(B)**. Nonmetric multidimensional scaling (NMDS) of rhizosphere microbiota of the flue-cured tobacco. **(C)**. Composition and distribution of rhizosphere microbiota of flue-cured tobacco at the phylum level. **(D)**. Composition and distribution of rhizosphere microbiota of flue-cured tobacco at the genus level.

Figure 2A shows that the alpha diversity index of the rhizosphere microbiota of flue-cured tobacco showed a decreasing trend with the increase in transplanting time (*p* < 0.05 or *p* < 0.01 or *p* < 0.001), indicating that the diversity and richness of the rhizosphere microbiota decreased gradually with the increase in transplanting time. The beta diversity index focuses on the comparison of diversity among different ecosystems, representing the differences among samples. In this study, nonmetric multidimensional scale analysis was used, which simplifies the data structure by reducing the dimension of the sample distance matrix to describe the distribution characteristics of samples at a specific distance scale. In the nonmetric multidimensional scale analysis chart (Figure 2B), the difference in soil microbial communities in the rhizosphere among the three groups was small, and the community composition was more similar. The comparison between groups shows that the composition of soil microbial communities in the rhizosphere at the three time points was quite different, indicating that the structure of soil microbial communities in the rhizosphere was significantly affected by the transplantation time.

### 3.3. Species composition and distribution of the flue-cured tobacco rhizosphere microbiota at different transplantation stages

The diversity of microbial communities in the rhizosphere soil of flue-cured tobacco at different transplantation stages was studied by means of high-throughput sequencing technology. Sequence alignment and annotation showed that at the phylum level, most of the rhizosphere microbiota in the three groups belonged to more than 30 phyla. Figure 2C (the first 10 phyla) shows that the dominant bacteria were Actinobacteria, Proteobacteria, Chloroflexi, Acidobacteria and Firmicutes. At the phylum level, the main microflora compositions of the three groups were similar. Further analysis found that the relative abundance of Firmicutes in the rhizosphere microbiota of flue-cured tobacco at the D60 transplantation time point increased significantly, while the relative abundance of Acidobacteria decreased significantly. At the D90 transplantation time point, the relative abundance of Actinobacteria in the rhizosphere microbiota of flue-cured tobacco decreased significantly, while the relative abundance of Proteobacteria increased significantly. At the genus level, the rhizosphere microbiota of the three groups covered more than 300 groups. Figure 2D (the first 20 genera) shows the structure and relative abundance of these three groups of microbiota. Dominant bacteria mainly include *Bacillus* sp., *Sphingomonas*, *Arthroactor*, *Saccharimonadales* and JG30-KF-AS9. Further analysis showed that the major genera of the two groups were similar at the genus level. The relative abundance of *Bacillus* sp. and *Arthrobacter* at the D60 transplantation time point increased significantly (*p* < 0.01 or *p* < 0.001), while the relative abundance of JG30-KF-AS9 decreased significantly. The relative abundance of *Sphingomonas* and *Saccharimonadales* at the D90 transplantation time point was significantly increased (*p* < 0.01 or *p* < 0.001), while the relative abundance of *Bacillus* sp. was significantly decreased compared with the D60 transplantation time point (*p* < 0.001).

### 3.4. Species differences and marker species of the flue-cured tobacco rhizosphere microbiota at different transplantation stages

**Figure 3:**
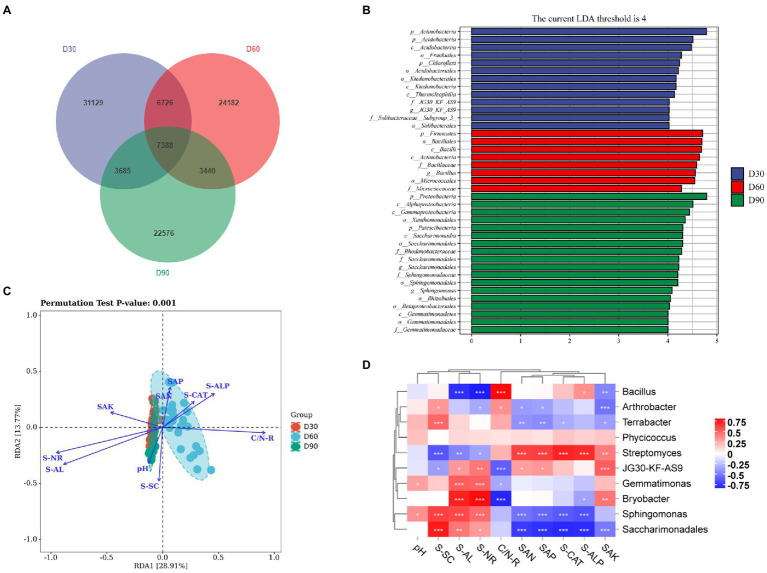
Species differences and marker species of the rhizosphere microbiota of flue-cured tobacco at different transplantation stages. **(A)**. Venn diagram of the rhizosphere microbiota of flue-cured tobacco. **(B)**. LEfSe analysis of rhizosphere microbiota of the flue-cured tobacco. **(C)**. RDA redundancy analysis between microbiota and physicochemical properties of the flue-cured tobacco rhizosphere soil. **(D)**. Correlation analysis between microbiota and physicochemical properties of the flue-cured tobacco rhizosphere soil.

The results show (Figure 3A) that there were 31,129, 24,182, and 22,576 ASV/OTUs in the D30, D60 and D90 groups of samples, respectively, and there were 7,388 ASV/OTUs in total in the three groups. The LDA value distribution histogram of species with significant differences in the LEfSe analysis was used to show the species significantly enriched in each group and their importance. The LEfSe analysis results showed that Actinomycetes, Firmicutes and Proteobacteria were significantly enriched and important species in the D30, D60 and D90 samples (Figure 3B). RDA results showed that SAN (soil available nitrogen content), SAP (soil available phosphorus content), S-CAT (soil catalase activity), S-ALP (soil available potassium content) and C/N-R (C/N ratio) were mainly related to the D60 group, and SAK (soil alkaline phosphatase activity), S-NR (soil nitrate reductase activity), S-AL (soil amylase activity), pH and S-SC (soil scrase activity) were mainly related to the D30 and D90 groups (Figure 3C). Online analysis software (https://www.OmicShare.com/Tools, Pearson correlation analysis) was used to analyze the correlation between the top 10 genera in the D30, D60 and D90 groups and soil physical and chemical property indices. The correlation analysis results show (Figure 3D) that microbial communities such as Terrabacter, Gemmatimonas, Bryobacter, Sphingomonas and Saccharimonadales in the rhizosphere soil of flue-cured tobacco were positively correlated with physical and chemical property indicators such as pH, S-SC, S-AL and S-NR in the rhizosphere soil of flue-cured tobacco but negatively correlated with physical and chemical property indicators such as C/N-R, SAN, SAP, S-CAT, S-ALP and SAK. In addition, Streptomyces had a significant positive correlation with physical and chemical indicators such as SAN, SAP, S-CAT, S-ALP, and SAK.

**Figure 4:**
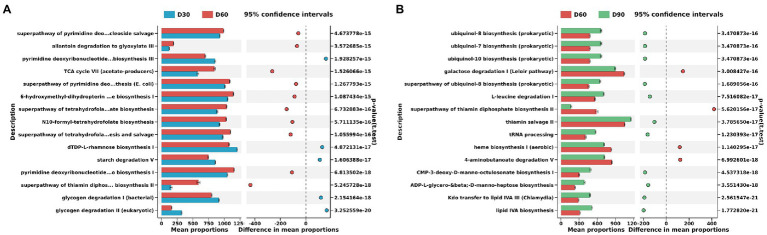
Differences in KEGG metabolic pathways of the flue-cured tobacco rhizosphere microbiota at different g transplantation stages. **(A)**. Differences in KEGG metabolic pathways of the flue-cured tobacco rhizosphere microbiota at the Day 30 and 60 transplantation stages. **(B)**. Differences in KEGG metabolic pathways of the flue-cured tobacco rhizosphere microbiota at the Day 60 and 90 transplantation stages.

### 3.5. Differences in metabolic pathways involved in the soil microbiota in the rhizosphere of flue-cured tobacco at different transplantation stages

The PICRUSt2 function prediction results showed that there were significant differences between the D30 and D60 flue-cured tobacco soil microbiota involved in metabolic pathways. D60 flue-cured tobacco soil microbiota significantly increased the superpathway of pyrimidine deoxyribonucleoside salvage, allantoin degradation to glyoxylate III and pyrimidine deoxyribonucleotides *de novo* biosynthesis III metabolic pathways (Figure 4A). Similarly, there was also a significant difference in the metabolic pathways involving the microbiota in the D60 and D90 flue-cured tobacco soils. The microbiota in the D90 flue-cured tobacco soils had significantly upregulated ubiquinol-8 biosynthesis (prokaryotic), ubiquinol-7 biosynthesis (prokaryotic) and ubiquinol-10 biosynthesis (prokaryotic) metabolic pathways (Figure 4B).

**Figure 5:**
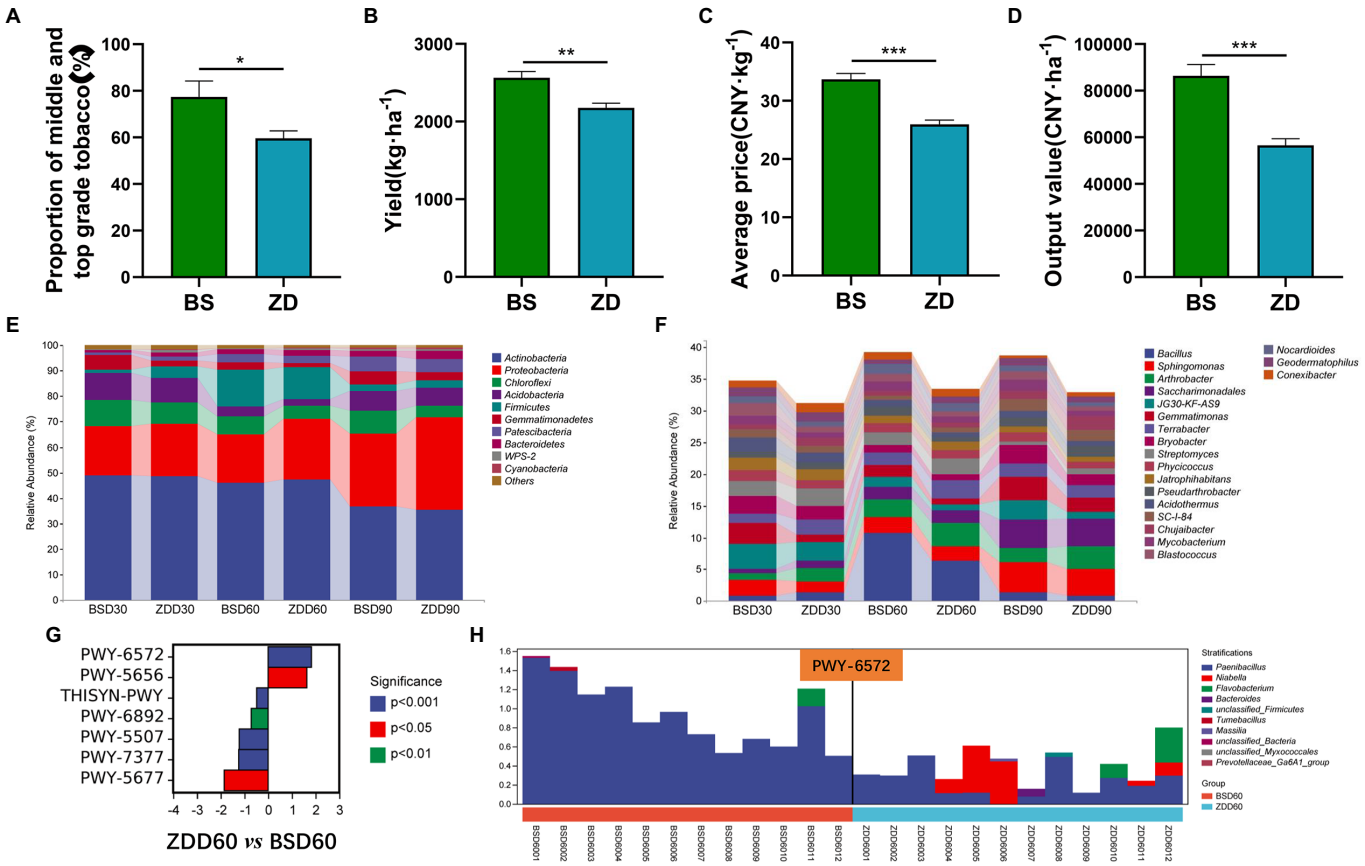
Differences in the flue-cured tobacco production characteristics and rhizosphere microbiota diversity in different regions. **(A)**. Proportion of middle- and top-grade flue-cured tobacco in different regions. **(B)**. Production of flue-cured tobacco in different regions. **(C)**. Average price of flue-cured tobacco in different regions. **(D)**. Output price of flue-cured tobacco in different regions. **(E)**. Composition and distribution of rhizosphere microbiota of flue-cured tobacco at the phylum level in different regions and stages. **(F)**. Composition and distribution of rhizosphere microbiota of flue-cured tobacco at the genus level in different regions and stages. **(G)**. Difference analysis of metabolic pathways involved in the rhizosphere microbiota of flue-cured tobacco in different regions at the Day 60 transplantation stage. **(H)**. Species composition and distribution of metabolic pathways (PWY-6572) involved in the rhizosphere microbiota of flue-cured tobacco in different regions at the Day 60 transplantation stage.

### 3.6. Differences in the flue-cured tobacco production characteristics and its rhizosphere microbiota diversity in different regions

The production characteristics of flue-cured tobacco in different regions were different. The proportion of middle and high grades, yield, average price and export value of flue-cured tobacco in the BS region were significantly higher than those in the ZD region (*p* < 0.05 or *p* < 0.01 or *p* < 0.001) (Figures 5A-D). In terms of microbial composition and distribution at the phylum level, the relative abundance of Proteobacteria in the BS region was significantly lower than that in the ZD region on the 60th day (*p* < 0.05), while the relative abundance of Firmicutes in the BS region was significantly higher than that in the ZD region on the 60th day (*p* < 0.05) (Figure 5E). At the genus level, the relative abundance of Bacillus in the BS region was significantly higher than that in the ZD region on the 60th day (*p* < 0.01) (Figure 5F). Further analysis was performed on the differences in metabolic pathways involving microbial communities in the rhizosphere soil of flue-cured tobacco in the BS and ZD regions on the 60th day (Figure 5G). The results showed that the PWY-6572 pathway was significantly upregulated in the BS region compared with the ZD region (*p* < 0.001), the PWY-6572 pathway was mainly contributed by *Paenibacillus*, and the relative abundance of *Paenibacillus* in the BS region was significantly higher than that in the ZD region (*p* < 0.01) (Figure 5H).

## 4. Discussion

The rhizosphere microbiome is a microorganism community designated to inhabit a narrow area (<10 mm) around the root system and plays an important role in maintaining crop health (Berendsen et al., 2012). In recent years, the important role of the rhizosphere microbiome in promoting the transformation of nutrient elements and inhibiting pathogens has been gradually revealed (Takenaka et al., 2008). There is an interaction between rhizosphere microorganisms and plant roots. The growth and reproduction of rhizosphere microorganisms provide nutrients for plant growth, while plant roots also provide a living environment for the growth of microorganisms, indirectly selecting and promoting the “survival of the fittest” rhizosphere microorganisms (Jouni et al.). Rhizosphere microorganisms can mineralize organic matter secreted by plant roots or in soil near roots and promote the circulation and turnover of nutrient elements (Alegria Terrazas et al., 2016). This promotes the release of nutrient elements in soil through the rhizosphere stimulation effect and affects the competitiveness of different plants (Bakker et al., 2018; Yin et al., 2018; Jat et al., 2019). In terms of plant disease control, beneficial microorganisms in the rhizosphere can directly act on pathogens through niche competition, production of antimicrobials, production of cell wall-degrading enzymes and other ways to reduce their abundance in the soil and can also directly act on plants themselves through production of plant growth hormone, fixation and decomposition of nutrient elements, induction of systemic resistance and population induction, thereby reducing the risk of plant disease (Liang et al., 2014; Wang L. et al., 2017; Ferreira et al., 2019). In this study, 16S rRNA high-throughput sequencing technology was used to analyze the differences in the rhizosphere microbiota of flue-cured tobacco in different transplant periods and different regions to provide a basis for improving the quality and performance and promoting the sustainable development of flue-cured tobacco in Southwest China.

The chemical properties of soil determine the growth of crops. There was no significant difference in the pH value of flue-cured tobacco rhizosphere soil at different transplanting stages, but there was a large difference in the C/N ratio of flue-cured tobacco rhizosphere soil at different transplanting stages. The activity of soil microorganisms changes the physical and chemical properties of soil and the soil microenvironment. The improvement of soil physical and chemical properties promoted the growth of tobacco plants and eventually led to changes in the growth and development of the tobacco. A high C/N ratio in soil will lead to the enrichment of fungi, and most of them will participate in the synthesis of antibiotics. Rhizosphere microbiota play a vital role in maintaining soil fertility and crop production (Carbone et al., 2014). The number of rhizosphere microbiota and the composition of the community structure will change with changes in the soil ecological environment (Hansel et al., 2008). Actinobacteria and Proteobacteria were the dominant bacteria in the rhizosphere soil of flue-cured tobacco at different transplanting stages. Actinobacteria can accelerate the decomposition of animal and plant remains in soil and accelerate the increase in inorganic content in soil. Proteobacteria mainly plays the role of nitrogen fixation. The increase in Proteobacteria is beneficial to the effective transformation of nitrogen in soil (Moulin et al., 2001). Moreover, the relative abundance of *Bacillus* in the rhizosphere of flue-cured tobacco at the D60 transplanting stage was significantly higher than that at the D30 and D90 transplanting stages. The change in the bacterial community (especially *Bacillus*, *Streptomyces* and *Sphingomonas*) in the rhizosphere soil of flue-cured tobacco is closely related to the content of available phosphorus, available potassium, organic matter and the soil C/N ratio. The physical and chemical properties of the flue-cured tobacco rhizosphere soil at different transplanting stages are different, resulting in different responses of soil microorganisms to different soil nutrients. In addition, the participation of rhizosphere microorganisms in the metabolic pathway of flue-cured tobacco in different transplanting periods was significantly different, mainly manifested in the superpathway of pyrimidine deoxyribonucleoside salvage, allentoin degradation to glycoxylate III and pyrimidine deoxyribonucleotides *de novo* biosynthesis III metabolic pathways, ubiquitol-8 biosynthesis (prokaryotic), ubiquinol-7 biosynthesis (prokaryotic) and ubiquinol-10 biosynthesis (prokaryotic) metabolic pathways.

The economic characteristics of flue-cured tobacco directly affect the income from tobacco leaves. Research shows that increasing the number of fungi, bacteria and Actinobacteria in the soil will improve the yield, output value, proportion of superior tobacco and average price of flue-cured tobacco to varying degrees (Sreevidya et al., 2016; Namwongsa et al., 2019). The yield, output value, average price and proportion of first-class tobacco in Boshang, Linxiang were significantly better than those of Zhangduo, Linxiang. The reason may be the difference in soil microbial communities between the two places. The relative abundance of Bacillus in the rhizosphere microbiota of flue-cured tobacco during D60 transplantation in Boshang, Linxiang, was significantly higher than that in Zhangduo, Linxiang. *Bacillus* contains many probiotics, such as *Bacillus subtilis*, *Lactobacillus lactis* and *Bacillus amylosus*. These microorganisms have strong probiotic functions, which can not only inhibit plant pathogens but also stimulate plant immunity to enhance the quality and performance of plants (Pieterse et al., 2014; Wu et al., 2019; Hu et al., 2020). Further analysis found that the rhizosphere microbiota of Linxiang Boshang flue-cured tobacco during the D60 transplantation had significantly upregulated PWY-6572 and PWY-5656 metabolic pathways (compared with the microbiota of the Linxiang Zhangduo area), with the PWY-6572 and PWY-5656 metabolic pathways being related to carbohydrate degradation and organic matter biosynthesis, respectively, which may be the key to promoting the quality and performance of Linxiang Boshang flue-cured tobacco to be better than that of the Linxiang Zhangduo area.

## 5. Conclusion

In this study, different transplanting periods of flue-cured tobacco had important effects on soil physical and chemical properties and rhizosphere microbial communities. There were significant differences in the rhizosphere microbiota and function of flue-cured tobacco in different regions, which may affect the performance and quality of flue-cured tobacco.

## Data availability statement

The datasets presented in this study can be found in online repositories. The names of the repository/repositories and accession number(s) can be found at: https://www.ncbi.nlm.nih.gov/genbank/, PRJNA921952.

## Author contributions

JW and YZ: data curation. YH: methodology. YX and JL: visualization. LZ: writing-original draft. LZ, SS, MF, SG, GL, WT, XD, and YH: writing-reviewing and editing. JD and FN: supervision, resources, funding acquisition. All authors contributed to the article and approved the submitted version.

## Funding

This research was funded by the science and technology projects of China Tobacco Corporation (110202201025 (LS-09)) and the science and technology projects of Yunnan Branch of China Tobacco Corporation (2020530000242017 and 2022530000241030).

## Conflict of interest

YH, YZ, YX, SS, MF, SG, GL, WT, and JW were employed by Lincang Branch Company of Yunnan Tobacco Company.

The remaining authors declare that the research was conducted in the absence of any commercial or financial relationships that could be construed as a potential conflict of interest.

## Publisher’s note

All claims expressed in this article are solely those of the authors and do not necessarily represent those of their affiliated organizations, or those of the publisher, the editors and the reviewers. Any product that may be evaluated in this article, or claim that may be made by its manufacturer, is not guaranteed or endorsed by the publisher.
